# Safety and Cost Analysis of Immunoglobulin Cessation Trials in Chronic Inflammatory Demyelinating Polyradiculoneuropathy

**DOI:** 10.1111/jns.70007

**Published:** 2025-02-18

**Authors:** Shiwen Koay, Yi‐Chun Chen, George Ransley, Laura Compton, Michael P. Lunn, Aisling S. Carr

**Affiliations:** ^1^ Queen Square Centre for Neuromuscular Diseases National Hospital for Neurology and Neurosurgery, UCLH London UK; ^2^ Department of Neuromuscular Diseases UCL Queen Square Institute of Neurology London UK

**Keywords:** cessation trials, chronic inflammatory demyelinating polyradiculoneuropathy, cost, intravenous immunoglobulin, safety

## Abstract

**Background and Aims:**

Chronic inflammatory demyelinating polyradiculoneuropathy (CIDP) is the most common chronic autoimmune neuropathy worldwide. A significant proportion of CIDP patients enter spontaneous or medication‐related remission, remaining stable without immunotherapy. Overtreatment of CIDP has clinical and financial implications. We examined performance of IVIg cessation trials in our CIDP cohort and report safety and cost analysis outcomes.

**Methods:**

In individuals with CIDP on maintenance IVIg treatment, a cessation trial was proposed in clinically stable patients with a static IVIg regimen over a 12‐month period. We explored the proportion who were stable off treatment for 12 or more months and the time to recovery in those who declined and were re‐treated. We examined cost implications of this approach.

**Results:**

45/125 individuals met criteria for clinical stability, with median age 58 years, I‐RODS 37/48, MRC‐SS 69/70 and annual treatment costs £107 000/person. Nine individuals had cessation trials resulting in decline within 2 years prior and were not re‐challenged, leaving 36 eligible individuals. 12 of 36 (33.3%) consented to cessation trial and eight of those (66.7%) remained stable off treatment for ≥ 12 months. The successful cessation trials resulted in a cost saving of £855 000/year, with a potential further saving of £1.7 million/year if all the eligible individuals had consented. All patients who deteriorated were rescued to previous baseline on retreatment.

**Interpretation:**

Individuals with CIDP should be counselled about the natural history of the disease and future scheduled, targeted cessation trials. A dedicated clinical infrastructure is vital to safely perform cessation trials.

## 1 Introduction

Chronic inflammatory demyelinating polyradiculoneuropathy (CIDP) is the most common chronic autoimmune neuropathy worldwide, with a prevalence of 3–5 individuals per 100 000 in the United Kingdom [1, 2]. Intravenous immunoglobulin (IVIg), steroids, and plasma exchange are evidence‐based first line treatments for CIDP, with 70%–80% of individuals showing a meaningful improvement in strength and function. Long‐term studies of individuals with CIDP suggest the disease may ‘burn out’ in a significant proportion of individuals, who remain stable without ongoing immunotherapy. In clinical trials where IVIg was withdrawn, 16%–55% of individuals did not deteriorate, indicating disease remission [3, 4].

Overtreatment of individuals with CIDP has important clinical and financial implications and constitutes a major challenge in CIDP trial design. From a safety perspective, the increased incidence of thromboembolic events in maintenance IVIg cohorts represents a meaningful clinical risk, particularly in the elderly and those with other cardiovascular risk factors [5, 6]. IVIg is a relatively scarce and expensive resource at a cost of £71/g, with immunomodulatory use representing £300 million per year of National Health Service (NHS) UK spending, and similar expenditure in other high income countries [7]. An equally important issue for the future of CIDP trials and management is the significant proportion of individuals in the placebo arm of clinical trials who do not relapse on IVIg withdrawal despite attempts in trial design to identify active disease in the screening process [8, 9].

The only current way to determine IVIg dependency (active disease controlled with maintenance IVIg) is to suspend or dose‐reduce treatment and observe for clinical deterioration. We previously retrospectively compared the outcomes of immediate treatment cessation and gradual dose reduction in a cohort of individuals with stable disease on maintenance IVIg. Twelve of 33 individuals (37.4%) remained stable without treatment for over 2 years, with a 50:50 split between immediate cessation and gradual dose reduction. In contrast to immediate treatment cessation the gradual dose reduction approach took between 0.91 and 5.87 years, with an average excess IVIg spend of £113 623 per individual. In those who did decline, documentation of disease activity was achieved by recording clinical outcome measures and all individuals who deteriorated were re‐stabilised to previous baseline, half within a week of restarting treatment [10]. Since 2019, our departmental approach has been to perform treatment cessation trials in the immediate cessation fashion in individuals with CIDP who have been clinically stable over 12 months on regular maintenance IVIg, with the consent of the patient and the support of a clinical nurse specialist and prompt access to senior neurologist assessment on the reporting of functional change. This approach is in keeping with UK National IVIg Commissioning Guidelines, which recommend annual assessment of individuals with CIDP on IVIg for evidence of clinical response, and periodic treatment cessation in those with stable disease [11]. A cessation challenge is often a psychologically difficult proposal for many individuals with CIDP, in whom IVIg often reversed significant disability and maintained independence for the duration of its use.

In this study, we examined our adherence to national guidance and the proportion of our IVIg‐maintenance CIDP cohort who were in disease remission with this approach. We present data on IVIg maintenance dose and clinical status before and after treatment cessation, with follow up of at least 12 months. We performed a cost analysis and developed a model to predict potential further cost savings if full compliance with national guidelines was achieved. Secondly, we performed a further review of individuals who consented to a cessation trial, with follow up of at least 20 months of those in disease remission, providing more complete data on longer‐term stability off treatment and a more comprehensive cost analysis in this cohort.

## 2 Materials and Methods

The study was conducted as part of routine clinical service evaluation with approval from the National Hospital for Neurology and Neurosurgery clinical governance committee. All CIDP individuals on maintenance IVIg treatment had a clinical review performed at least annually with disease specific outcome measures (most commonly the MRC‐SS, I‐RODS, grip strength and 10‐m walk), as well as review of IVIg tolerance, dose and regimen and consideration of cardiovascular risk factors.

Firstly, we identified individuals on a stable IVIg maintenance dosing regimen (g/kg/month) with no dose changes over at least 12 months between January 2021 and June 2023. Secondly, we identified clinical stability by excluding individuals with greater than minimally clinically important difference (MCID) in one outcome measure or contiguous sub‐MCID deterioration in two or more outcome measures over that timescale. We omitted any individual who had a cessation trial performed within the preceding 3 years (2018–2021) that had revealed active disease, with documentation of greater than MCID in one outcome measure or contiguous sub‐MCID deterioration in two or more outcome measures on treatment cessation.

In this clinically stable cohort of CIDP individuals, we noted demographic and clinical features, IVIg dose, and days spent on our daycare unit. We noted the proportion of eligible individuals who underwent treatment cessation trials, and of these, the proportion who deteriorated upon treatment withdrawal and those who remained stable off treatment for at least 6 months. For the financial modelling, we calculated the average unit price for 13 commercially available IVIg products (£70.84), the cost of day care unit delivery of IVIg infusion (£384 per patient/day including overhead costs, consumables, and medical, nursing, and administrative staffing). This was compared against the cost of running our immunosuppression service, which includes a weekly consultant‐led immunosuppression clinic (£16 744 per annum) and a full‐time band 7 clinical nurse specialist (£43 742 per annum). Statistical analysis was performed on STATA/MP 17. Summary data is presented as median (inter‐quartile range). Mann–Whitney tests were used to compare groups. Two‐tailed *p*‐values < 0.05 were considered significant.

## 3 Results

### 3.1 Study Population

A total of 125 individuals with CIDP were on maintenance IVIg treatment, with 76 on a stable dose over 12 months. Clinical outcome measurements were documented in 66 individuals at two timepoints at least 12 months apart during the study period. Of these 66 individuals, 45 (68.2%) met criteria for clinical stability (Table 1). In this subset, the median age was 58 years (IQR 52–70 years), and 56% were male. The median cost of IVIg was £97 705 per person/year, at a median frequency of 2.4 days/month in the day care unit (£9213 per person/year), giving a total median cost of £106 918 per person/year.

**TABLE 1:** Clinically stable CIDP Cohort.

Demographic and clinical details	
Total number	45
Male sex, *n* (%)	25 (56%)
	Median (IQR)
Age, year	58 (52–70)
Disease duration, year	8.5 (6–14)
I‐RODS	36 (25–43)
MRC‐SS	69 (64–70)
Annual cost, £, per person/year	
IVIg	97 705 (66 408–123 275)
Daycare delivery	9213 (4606–13 819)
Total median annual cost, per person/year	107 000 (71 000–137 100)

### 3.2 Treatment Cessation

**FIGURE 1:**
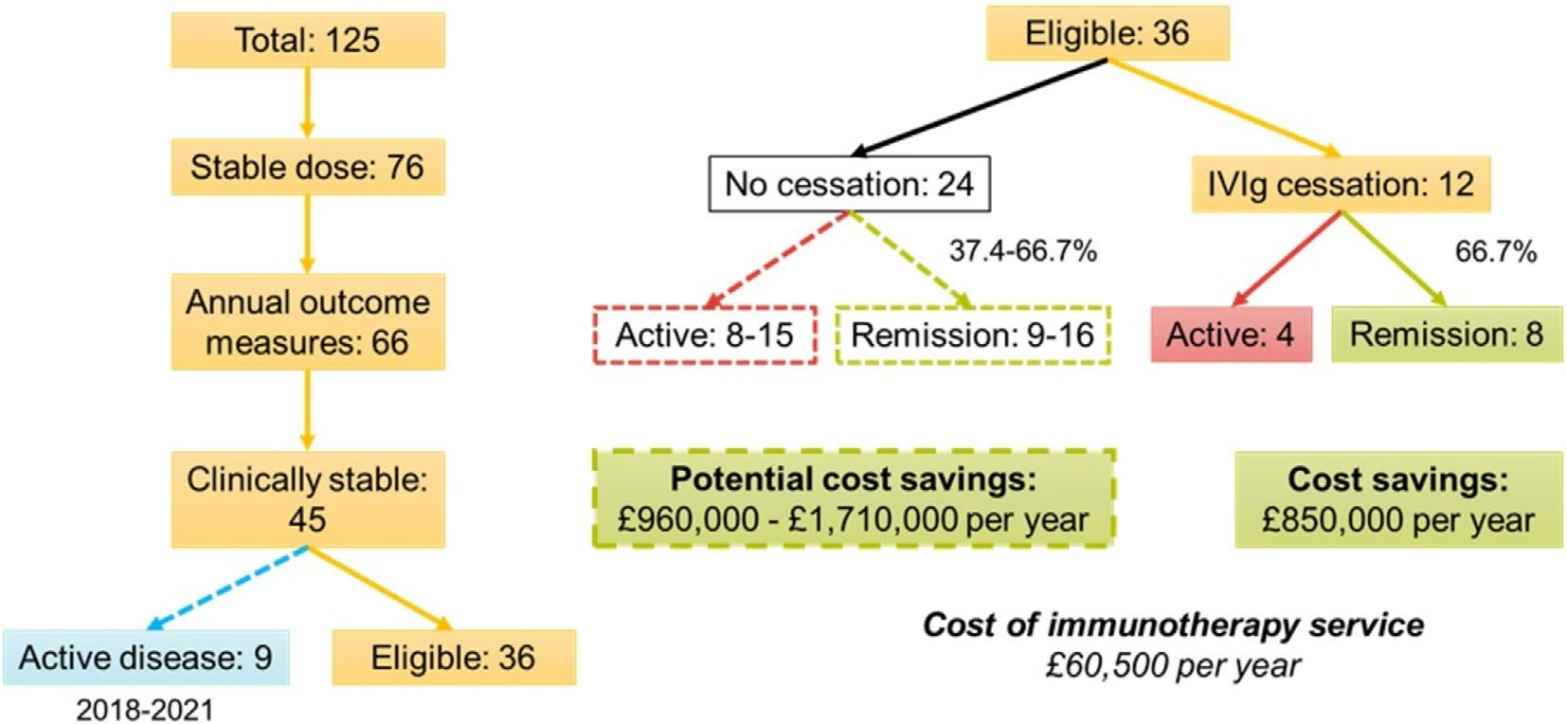
Financial impact of IVIg cessation trials in CIDP. Flowchart illustrating individuals with CIDP with stable disease, proportion challenged, financial impact of successful cessation trials and potential cost savings if all eligible patients consented to treatment cessation trials.

Of these 45 individuals, nine had undergone recent treatment cessation trials in the 3 years prior to the study, with clinically significant deterioration documented, indicating active disease (Figure 1). These individuals had been destabilized on IVIg and not re‐challenged during this study period, leaving 36 eligible individuals. Twelve of 36 (33.3%) consented to a treatment cessation trial which was performed. Eight of those 12 individuals (66.7%) remained stable off treatment for at least 12 months. Within the clinically stable cohort, three individuals were on additional immunosuppression with mycophenolate mofetil (1.5 g BD). Of these, one underwent a treatment cessation trial and remained stable off IVIg on mycophenolate alone.

We explored differences in groups who underwent cessation trials and did not, and those who remained stable or relapsed. The MRC‐SS was slightly higher (better) in the treatment cessation group (median 70, IQR 66–70) compared to the group who did not consent to a treatment cessation trial (median 68, IQR 63–70, *p* = 0.06), however I‐RODS scores were similar. There were no significant clinical, demographic or IVIg dosing differences between the groups (Table S1). Amongst the group who had a cessation trial, the eight individuals who remained stable off IVIg had a slightly shorter disease duration (median 7 years, IQR 6–11 years) compared to the group who had active disease (median 14 years, IQR 12–16 years), although this difference was not significant (*p* = 0.06). No other significant differences were found between the IVIg dependent (active disease) group and those with inactive disease (Table S2).

In the individuals with demonstrated active disease, median time to deterioration on IVIg cessation was 17 weeks (IQR 14–23 weeks) with median fall in MRC‐SS of 2 points (IQR 0–4) and change in I‐RODS of 12 (IQR 11–13). All individuals re‐achieved their previous baseline (Figures S1 and S2). Three individuals (Patient A, C and D) were treated with an induction regimen (2 g/kg/month for 1–3 cycles) and reached their baseline within 4–15 weeks [12]. One individual (Patient B) resumed their normal IVIg regimen (1.5 g/kg/month), reaching their previous baseline more slowly but within 28 weeks.

### 3.3 Financial Model

The cost saving associated with successful IVIg cessation in eight individuals was £855 344 per year, including IVIg spend and daycare unit costs. This is over 14 times the cost of providing the immunosuppression service at our hospital. If we consider an IVIg cessation success rate of between 37.4% and 66.7%, based on observed rates in this study and previously published rates in our cohort [7], and apply this to the remaining 24 eligible individuals in whom a cessation trial was not performed during this period, we expect to reveal another 9–16 individuals in remission. This would represent a further potential annual cost saving of £962 262–£1 710 688 per year.

### 3.4 Longer‐Term Follow Up and Financial Evaluation

We subsequently reviewed the 8 patients who did not deteriorate in our study when treatment was withdrawn at a second time‐point to evaluate longer term stability. All but one patient have continued to remain stable off treatment to date up until December 2024. This patient noticed deterioration with finger drop after 13 months off IVIg, with neurophysiology that showed a deterioration in radial motor action potential which has previously been affected and improved with prior IVIg treatment. He returned to his usual regimen with return to baseline clinically and neurophysiologically.

Overall, the cessation trials in 12 patients resulted in stopping treatment for seven patients that have now remained stable for at least 20 months (median 34 months, IQR 22–37 months), resulting in a cost saving of £1 734 067 to date. A total of five patients deteriorated off treatment and resumed IVIg with a median pause in treatment of 7.3 months (IQR 7.3–11.3 months). Two of these patients resumed their normal IVIg regimen, and three patients had bolus treatment of 2 g/kg for 1–3 doses at an additional cost of £28 397 compared to their usual regimen. The cost saved from the temporary IVIg cessation in these five patients was £282 567, considering the cost of the boost regimen. The overall savings from the cessation trials conducted to date in this cohort is £2 016 634.

## 4 Discussion

We examined a real‐life clinical practice approach to the assessment of disease activity in individuals with CIDP treated with maintenance IVIg and demonstrate the financial implications of this approach. IVIg is a relatively scarce and clearly expensive resource. In 2021–2022, CIDP accounted for the second highest IVIg use within the UK (1.27 million grams at a cost of £89.5 million/year) [7]. In this real life study, two‐third of individuals with CIDP and stable disease on maintenance IVIg who consented to a cessation trial were found to be in remission on withdrawal of treatment for at least 12 months, resulting in an annual cost saving of £850 000 per year, and a potential further annual saving of up to £1.7 million per year if all eligible individuals had consented and remissions were maintained. There were no clinical, demographic or IVIg dosing differences between those who remained stable after treatment withdrawal and those who deteriorated revealing active disease. All those who deteriorated were able to re‐establish previous baseline on re‐treatment. Despite an agreed departmental consensus approach to the performance of cessation trials, only 12 of 36 eligible individuals in this cohort had a cessation trial performed. Further exploration is required to understand the reasons behind this discrepancy, but a significant proportion did not consent, and we could not mandate treatment suspension. This approach to IVIg cessation in CIDP is safe to individuals but is dependent on a skilled clinical infrastructure to facilitate it. Nevertheless, the cost benefit is significant and the risk benefit probably favorable.

Whilst there are established arguments for IVIg withdrawal trials in CIDP, which form part of national and international consensus guidelines, there is a large variation in whether and how these are performed, influenced by previous experience of individual clinicians and the infrastructure available to monitor and support patients though a treatment withdrawal process. In this study, we examine our own practice in a large national neuromuscular center in the UK, calculate the cost implications and discuss how we could potentially improve upon current performance compared to national guidance. In patients with CIDP who have stable disease on maintenance IVIg treatment, we feel that stopping treatment directly in those who consent is preferable for several reasons. Firstly, compared to immediate treatment cessation, a gradual dose reduction is associated with a longer time to demonstrate treatment dependence/active disease (mean time to deterioration 8.8 vs. 3.5 months in our previous audit), with an excess IVIg spend of £113 623 per individual [10]. Secondly, a tapered treatment withdrawal regimen may be more acceptable to some patients but can result in a longer period of psychological vigilance. A recent placebo‐controlled double‐blinded RCT trial where half of patients were randomized to continue IVIg, and half had a gradual reduction in IVIg dose over 3 cycles with increasing volumes of placebo infusion followed by placebo only infusions found that amongst the 31 patients allocated to continue treatment, 13 individuals 42% reached pre‐defined relapse end‐point, due to a MCID change in CIDP outcome measures (*n* = 5), or patient (*n* = 5) or physician (*n* = 3) decision regarding likely relapse in the absence of MCID change [3]. Furthermore, previous latent class modelling using 1400 data points in 100 patients with inflammatory neuropathies over 10 years at a single neuroscience center previously showed intra‐individual variation of CIDP specific outcome measures is common during periods of clinical stability, often exceeding MCID [13]. An immediate cessation trial may avoid a prolonged period of psychological vigilance for potential decline and can facilitate clear capture of objective deterioration above and beyond the ‘noise’ seen with serial repetition of outcome measures over several months.

We acknowledge the anxiety, for both patient and clinicians, associated with treatment withdrawal and the potential for functional deterioration in a relapsing‐ remitting disease. A relatively small number of patients ultimately consented to a treatment withdrawal in the time period studied, highlighting the real‐life difficulties of conducting cessation trials even within a national center with a dedicated consultant‐led inflammatory neuropathy service and clinical nurse specialist support. Patients often recall the huge impact that IVIg treatment made initially on their disease, in some cases allowing them to go from being bed bound to back to normal function within weeks and are naturally nervous about a potential decline back to pre‐treatment disability. Without any reliable predictors of individuals with active disease we use clinical stability over at least 12 months on stable IVIg dosing regimens to identify eligible patients and quote an approximate 40% likelihood of remission off treatment to all. It is not uncommon for multiple discussions to reiterate the rationale for cessation trials and obtain informed consent prior to the performance of each closely supported treatment cessation attempt. Furthermore, our study was conducted between 2021 and 2023, when clinical practice was affected by the COVID‐19 pandemic and its aftermath, with greater difficulties in arranging face‐to‐face consultations impacting on our ability to perform objective physical assessments using CIDP specific outcome measures. Additionally, patients who have had a previous decline when treatment was withdrawn and subsequently resume treatment with destabilization are often even more reticent about further cessation trials, even after a period of stability so we withhold further cessation attempts if there was clear demonstration of active disease within the previous 2–3 years. Where appropriate, we sometimes consider adding an IVIg‐sparing immunomodulatory treatment such as mycophenolate mofetil or azathioprine, prior to attempting to titrate down or subsequently stop IVIg.

CIDP is probably not a single disease but an umbrella term encompassing a spectrum of treatable demyelinating neuropathies with an inflammatory pathogenesis, with patients variably demonstrating monophasic, relapsing or more chronic disease course [2]. IVIg also has multiple mechanisms of action, and individual patients often demonstrate a variable degree of responsiveness in the speed and extent of recovery with treatment and decline on withdrawal of treatment. There is currently no consensus on when patients are defined to be in disease remission when taken off treatment, and different observational studies and RCTs in CIDP have used different study designs making them difficult to compare directly. In the PATH study, which was designed to compare SCIg to placebo, the study design incorporated an IgG dependence phase when patients on IVIg treatment had decline when treatment was withdrawn within a 12‐week period in order to be eligible for the study. 207/245 patients (85%) were found to relapse within this time frame. In contrast, in our real‐life departmental audits on IVIg cessation, the median time to deterioration when IVIg was withdrawn was 3.6–3.9 months, more comparable to the 4.5 months seen in the study from Nobile‐Orazio et al. comparing time to relapse after withdrawal of IVIg and corticosteroids [14]. Overall, it is likely most patients will relapse within months of withdrawing IVIg, but a smaller number of patients may initially remain stable for a more prolonged period before relapsing, at least within the limits of current clinical outcome measures. We must offer robust and reliable care models to reflect the variability in our patient cohorts. All patients undergoing cessation trials in our center are given the details of our clinical nurse specialist to contact if they experience a decline in their symptoms, so they can be promptly assessed and re‐initiated on treatment if appropriate. We advocate booking routine follow‐up 6 months after stopping IVIg, and if patients are stable at this stage, offering a further follow up appointment at 6 or 12 months. After a longer period of stability, when patients may be appropriate for discharge from a specialist clinic, we provide a safety net mechanism of patient initiated follow up, where patients can contact our specialist nurse (LC) to book a review without having to see their general practitioner to obtain a referral back to our service.

We advocate using multiple disease specific outcome measures where possible to get a better understanding of disease trajectory that is tailored to individual patients. We are currently working on building a disease monitoring tool in our electronic healthcare record system to allow us to track multiple different outcome measures alongside treatment schedules, with a graphical representation to facilitate clinician and patient understanding of disease status and inform discussions regarding potential treatment cessation or escalation, if indicated. Furthermore, clinicians must consider other common causes of a functional deterioration in mobility and gait other than active CIDP in an aging population, including concomitant musculoskeletal disease, radiculopathy, idiopathic Parkinson's disease and other systemic illnesses.

Whilst there were no significant differences between groups, individuals who consented to treatment trials had a slightly higher MRC‐SS than individuals who did not. Individuals with fixed disability due to previous axonal loss may be more reluctant to proceed with a treatment cessation trial through fears of further decline. Patient understanding and engagement is essential to conduct and accurately assess disease activity during cessation trials. When making an initial diagnosis of CIDP, it is important to counsel individuals about the natural history of the disease, including initiation of treatment, evaluation of treatment response and disease stability, with subsequent treatment cessation where appropriate. In individuals on long‐term IVIg treatment, who may not be familiar with the potential for disease remission, this information should be discussed at annual review appointments. As thromboembolic risk with regular IVIg treatment increases with age and the presence of cardiovascular risk factors, it is increasingly important to explore IVIg dependence as an individual ages.

To safely perform IVIg cessation trials, a robust clinical infrastructure is needed to facilitate prompt assessment of individuals who deteriorate and re‐initiate treatment when necessary. We run a specialist inflammatory peripheral nerve service with the support of a dedicated immunosuppression specialist nurse and one consultant‐led clinic per week. This clinical infrastructure is vital for patient engagement in the process, which can be psychologically challenging because of the unpredictable risk of functional decline. The cost of providing this service is substantially lower than the cost savings from successfully withdrawing IVIg treatment in a proportion of clinically stable individuals. Given the prevalence of CIDP, a population of 2–3 million is likely to support a similar system to this, on the basis of 100–150 prevalent cases. The UK NHSE IVIg Commissioning Guidelines reflect international consensus guidelines on CIDP diagnosis, management and monitoring, incorporating evidence‐based and consensus‐based recommendations for good clinical practice [15]. Annual review of disease activity using clinimetrically validated, disease specific outcome measures allows clinicians to identify patients suitable for treatment cessation trials and demonstrate ongoing need for treatment. Clinicians who treat individuals with CIDP should be supported to enable them to conduct IVIg cessation trials in appropriate individuals in a safe clinical infrastructure. We hope this study provides data which will help facilitate safe, appropriate and responsible use of IVIg in CIDP.

## Conflicts of Interest

Michael P. Lunn has given advice on ad hoc advisory boards particularly on trial design to Roche, AstraZeneca, Sanofi, UCB, Sanofi, Takeda, Polyneuron and BeiGene (conference expenses and advisory board). Unrestricted speaker fees for Beigene and Grifols for the production of educational materials. Unrestricted conference expenses have been received from Beigene and CSL Behring. He has received research grants from Charitable Foundations: Patrick Berthoud Charitable Trust, ABN, Guarantors of Brain, National Brain Appeal, UCLH Charities, Leonard Wolfson Foundation, Medical Research Council, GBS CIDP Foundation International, New Zealand Medical Foundation, GAIN UK. Aisling S. Carr has given advice on ad hoc advisory boards for Takeda, Grifols and CSL. Unrestricted speaker fees for Beigene, Takeda, Alnylam, Grifols and BMS for the production of educational materials. Unrestricted conference expenses have been received from Takeda and CSL Behring. She has received research grants from Charitable Foundations: Guarantors of Brain. Shiwen Koay has received research grants from Charitable Foundations: Guarantors of Brain and National Brain Appeal.

## Supporting information


**Table S1.** Comparison of individuals who consented to treatment cessation trials and who were not challenged.

**Table S2.** Comparison of individuals with active disease who relapsed without treatment and individuals remaining stable off IVIg for at least 12 months.

**Figure S1.** I‐RODS at baseline, at deterioration following IVIg withdrawal, and at recovery following reinstatement of IVIg.
**Figure S2.** MRC‐SS at baseline, at deterioration following IVIg withdrawal, and at recovery following reinstatement of IVIg.

## Data Availability

The data that support the findings of this study are available from the corresponding author upon reasonable request.

## References

[1] Y. A. Rajabally , B. S. Simpson , S. Beri , J. Bankart , and J. A. Gosalakkal , “Epidemiologic Variability of Chronic Inflammatory Demyelinating Polyneuropathy With Different Diagnostic Criteria: Study of a UK Population,” Muscle & Nerve 39, no. 4 (2009): 432–438. 19260065 10.1002/mus.21206

[2] M. Mahdi‐Rogers and R. A. Hughes , “Epidemiology of Chronic Inflammatory Neuropathies in Southeast England,” European Journal of Neurology 21, no. 1 (2014): 28–33. 23679015 10.1111/ene.12190

[3] M. E. Adrichem , I. M. Lucke , A. Vrancken , et al., “Withdrawal of Intravenous Immunoglobulin in Chronic Inflammatory Demyelinating Polyradiculoneuropathy,” Brain 145, no. 5 (2022): 1641–1652. 35139161 10.1093/brain/awac054 PMC9166547

[4] M. E. Adrichem , F. Eftimov , and I. N. van Schaik , “Intravenous Immunoglobulin Treatment in Chronic Inflammatory Demyelinating Polyradiculoneuropathy, a Time to Start and a Time to Stop,” Journal of the Peripheral Nervous System 21, no. 3 (2016): 121–127. 27241239 10.1111/jns.12176

[5] M. Kapoor , J. Spillane , C. Englezou , et al., “Thromboembolic Risk With IVIg: Incidence and Risk Factors in Patients With Inflammatory Neuropathy,” Neurology 94, no. 6 (2020): e635–e638. 31852814 10.1212/WNL.0000000000008742 PMC7136065

[6] M. Kapoor , I. Hunt , J. Spillane , et al., “IVIg‐Exposure and Thromboembolic Event Risk: Findings From the UK Biobank,” Journal of Neurology, Neurosurgery, and Psychiatry 93, no. 8 (2022): 876–885. 35688633 10.1136/jnnp-2022-328881

[7] M. Foster , “NHS Immunoglobulin Database Annual Report 2021/2022,” (2023), https://igd.mdsas.com/wp‐content/uploads/Igd_DataReport_202122.pdf.

[8] I. N. van Schaik , V. Bril , N. van Geloven , et al., “Subcutaneous Immunoglobulin for Maintenance Treatment in Chronic Inflammatory Demyelinating Polyneuropathy (PATH): A Randomised, Double‐Blind, Placebo‐Controlled, Phase 3 Trial,” Lancet Neurology 17, no. 1 (2018): 35–46. 29122523 10.1016/S1474-4422(17)30378-2

[9] R. A. Lewis , D. R. Cornblath , H. P. Hartung , et al., “Placebo Effect in Chronic Inflammatory Demyelinating Polyneuropathy: The PATH Study and a Systematic Review,” Journal of the Peripheral Nervous System 25, no. 3 (2020): 230–237. 32627277 10.1111/jns.12402 PMC7497019

[10] M. Kapoor , L. Compton , A. Rossor , et al., “An Approach to Assessing Immunoglobulin Dependence in Chronic Inflammatory Demyelinating Inflammatory Polyneuropathy,” Journal of the Peripheral Nervous System 26, no. 4 (2021): 461–468. 34637194 10.1111/jns.12470

[11] N. England , Clinical Commissioning Policy for the Use of Therapeutic Immunoglobulin (Ig) England (NHS England, 2024).

[12] M. P. Lunn , L. Ellis , R. D. Hadden , Y. A. Rajabally , J. B. Winer , and M. M. Reilly , “A Proposed Dosing Algorithm for the Individualized Dosing of Human Immunoglobulin in Chronic Inflammatory Neuropathies,” Journal of the Peripheral Nervous System 21, no. 1 (2016): 33–37. 26757367 10.1111/jns.12158

[13] R. Y. S. Keh , D. A. Selby , S. Jones , et al., “Predicting Long‐Term Trends in Inflammatory Neuropathy Outcome Measures Using Latent Class Modelling,” Journal of the Peripheral Nervous System 27, no. 1 (2022): 84–93. 34936164 10.1111/jns.12481

[14] E. Nobile‐Orazio , D. Cocito , S. Jann , et al., “Frequency and Time to Relapse After Discontinuing 6‐Month Therapy With IVIg or Pulsed Methylprednisolone in CIDP,” Journal of Neurology, Neurosurgery, and Psychiatry 86, no. 7 (2015): 729–734. 25246645 10.1136/jnnp-2013-307515

[15] P. Y. K. Van den Bergh , P. A. van Doorn , R. D. M. Hadden , et al., “European Academy of Neurology/Peripheral Nerve Society Guideline on Diagnosis and Treatment of Chronic Inflammatory Demyelinating Polyradiculoneuropathy: Report of a Joint Task Force‐Second Revision,” European Journal of Neurology 28, no. 11 (2021): 3556–3583. 34327760 10.1111/ene.14959

