# Supplementary material for: Safety and Cost Analysis of Immunoglobulin Cessation Trials in Chronic Inflammatory Demyelinating Polyradiculoneuropathy
Source: J Peripher Nerv Syst. 2025 Feb 18;30(1):e70007. doi: 10.1111/jns.70007 (PMC11836592; doi:10.1111/jns.70007)
Supplement: Supplementary file 1 — Table S1. Comparison of individuals who consented to treatment cessation trials and who were not challenged. [file JNS-30-0-s003.docx]

Supplementary Table 1: Comparison of individuals who consented to treatment cessation trials and who were not challenged

|  | Not challenged | Treatment cessation | *P*-value |
| --- | --- | --- | --- |
| Total number | 24 | 12 |  |
| Male sex | 12, 50% | 7, 58% | 0.64 |
|  | Median, IQR | |  |
| Age, y | 57, 47-62 | 61, 50-71 | 0.26 |
| Disease duration, y | 7, 5-10 | 12, 6-14 | 0.34 |
| I-RODS | 33, 25-39 | 33, 29-38 | 0.69 |
| MRC-SS | 68, 63-70 | 70, 67-70 | 0.06 |
| IVIg dose, g/kg/month | 1.7,1.4-2.5 | 1.4,1.2-1.7 | 0.13 |
| Daycare unit days/month | 2.7, 2.0-4.0 | 2.0, 1.3-3.2 | 0.14 |
| MMF | 2, 8% | 1, 8% | 1 |
