# Supplementary material for: Safety and Cost Analysis of Immunoglobulin Cessation Trials in Chronic Inflammatory Demyelinating Polyradiculoneuropathy
Source: J Peripher Nerv Syst. 2025 Feb 18;30(1):e70007. doi: 10.1111/jns.70007 (PMC11836592; doi:10.1111/jns.70007)
Supplement: Supplementary file 2 — Table S2. Comparison of individuals with active disease who relapsed without treatment and individuals remaining stable off IVIg for at least 12 months. [file JNS-30-0-s002.docx]

Supplementary Table 2: Comparison of individuals with active disease who relapsed without treatment and individuals remaining stable off IVIg for at least 12 months.

|  | Active disease | Stable off IVIg | *P*-value |
| --- | --- | --- | --- |
| Total number | 4 | 8 |  |
| Male sex | 3/4 (75%) | 4/8 (50%) | 0.42 |
|  | Median, IQR | |  |
| Age, y | 61, 52-66 | 64, 53-71 | 0.79 |
| Disease duration, y | 14, 12-16 | 7, 6-11 | 0.06 |
| I-RODS | 27, 22-31 | 36, 33-45 | 0.11 |
| MRC-SS | 68, 65-70 | 70, 70-70 | 0.14 |
| IVIg dose, g/kg/month | 1.4, 1.2-1.5 | 1.6,1.3-2.0 | 0.48 |
| DCU days/month | 2.0, 1.3-2.8 | 2.0, 1.4-3.0 | 0.84 |
| MMF | 0, 0% | 1, 8% | 1 |
