# Supplementary figures and images for: Safety and Cost Analysis of Immunoglobulin Cessation Trials in Chronic Inflammatory Demyelinating Polyradiculoneuropathy
Source: J Peripher Nerv Syst. 2025 Feb 18;30(1):e70007. doi: 10.1111/jns.70007 (PMC11836592; doi:10.1111/jns.70007)

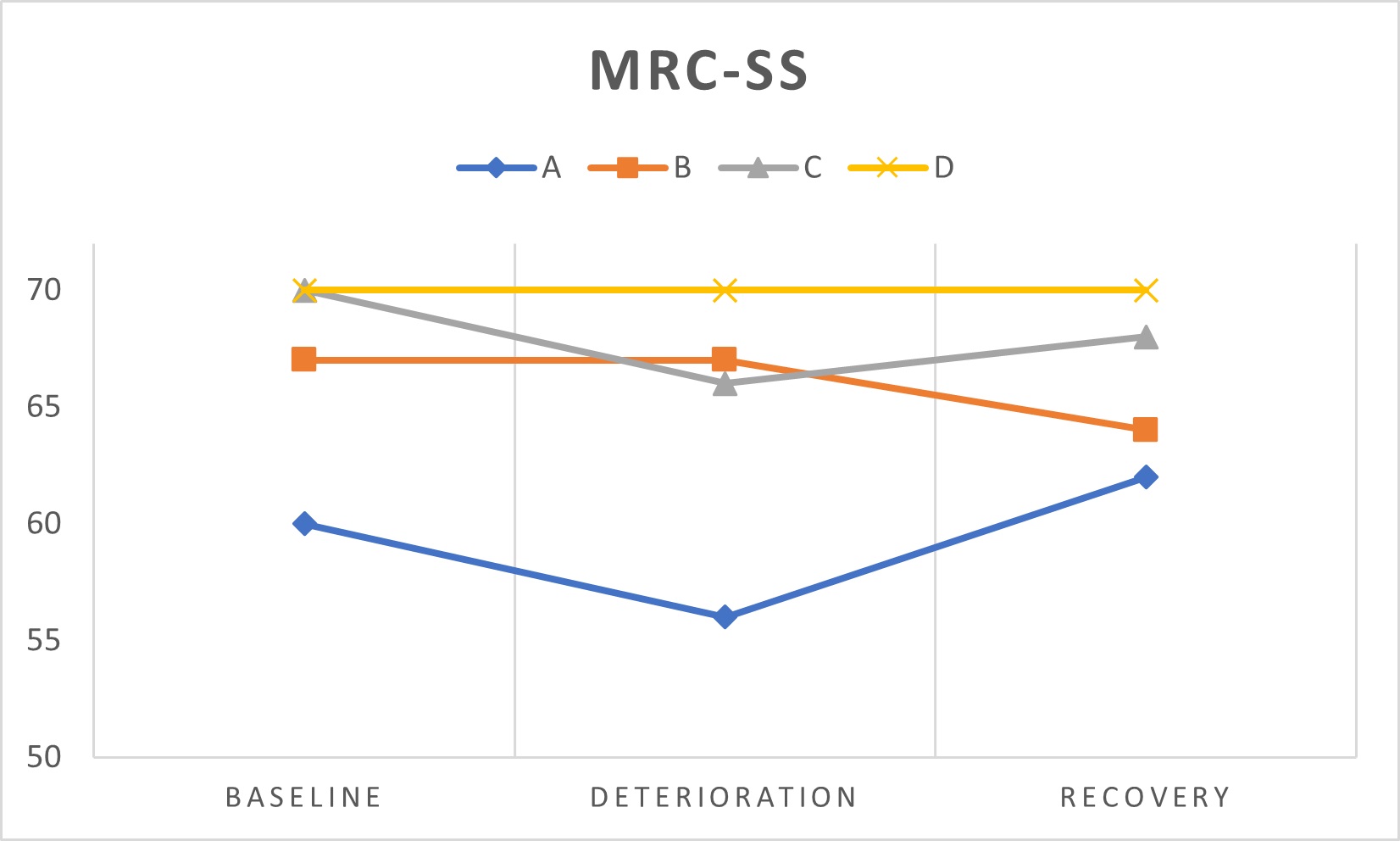

Supplement: Supplementary file 3 — Figure S1. I‐RODS at baseline, at deterioration following IVIg withdrawal, and at recovery following reinstatement of IVIg. Figure S2. MRC‐SS at baseline, at deterioration following IVIg withdrawal, and at recovery following reinstatement of IVIg. [file JNS-30-0-s001.zip › 6. Supplementary Figure 2_MRC-SS.jpg]

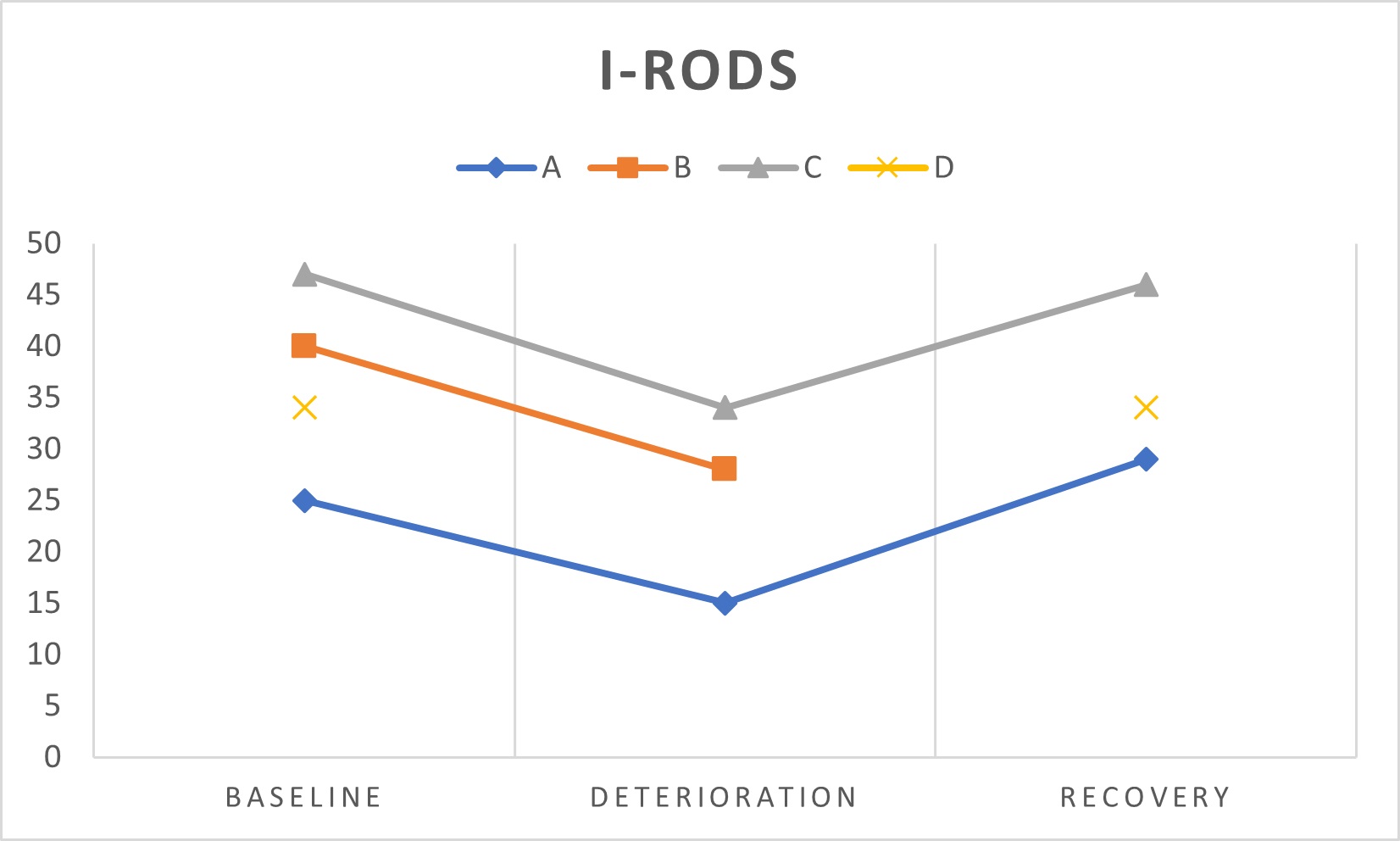

Supplement: Supplementary file 3 — Figure S1. I‐RODS at baseline, at deterioration following IVIg withdrawal, and at recovery following reinstatement of IVIg. Figure S2. MRC‐SS at baseline, at deterioration following IVIg withdrawal, and at recovery following reinstatement of IVIg. [file JNS-30-0-s001.zip › 5. Supplementary Figure 1_I-RODS.jpg]
